# Prediction of Higher Ki-67 Index in Pituitary Adenomas by Pre- and Intra-Operative Clinical Characteristics

**DOI:** 10.3390/brainsci12081002

**Published:** 2022-07-28

**Authors:** Xuanzhi Wang, Mingwu Li, Xiaofeng Jiang, Fei Wang, Shiying Ling, Chaoshi Niu

**Affiliations:** 1 Department of Neurosurgery, The First Affiliated Hospital of USTC, Division of Life Sciences and Medicine, University of Science and Technology of China, Hefei 230001, China; wangxuanzhi2013@163.com (X.W.); lmw3014@sohu.com (M.L.); xfjiang110@126.com (X.J.); neurosurgeonahwf@126.com (F.W.); lingshiying1@126.com (S.L.); 2 Anhui Province Key Laboratory of Brain Function and Brain Disease, Hefei 230001, China; 3 Anhui Provincial Clinical Research Center for Neurosurgical Disease, Hefei 230001, China

**Keywords:** influence factor, Ki-67, pituitary adenoma, blood supply, tumor aggression

## Abstract

Objective: The Ki-67 index is an indicator of the active proliferation and aggressive behavior of pituitary adenomas (PAs). Appropriate pre- and intra-operatives of the Ki-67 index can help surgeons develop better and more personalized treatment strategies for patients with PAs. This study aimed to investigate the influence factors for predicting the Ki-67 index in PAs. Methods: Data of 178 patients with PAs confirmed by pathology were retrospectively analyzed. According to the Ki-67 index, the patients were divided into the Ki-67 < 3% and Ki-67 ≥ 3% cohorts. Patient data, including age, sex, postoperative immunohistochemical pituitary hormone positive index, Knosp grade, tumor breaking through the sellar floor, rich blood supply to the tumor, tumor located inside the sella, erosion of the dorsum sellae bone, and pituitary-specific transcription factor, were collected. A univariate logistic analysis was used to evaluate the influence factors for a high Ki-67 index. Multiple regression and receiver operating characteristic (ROC) curve were used to analyze the factors with *p* < 0.05. The mutant status of Ki-67 index was predicted by nomogram. Results: Multivariate regression analysis showed that rich blood supply to the tumor and erosion of the dorsum sellae bone were independent risk factors for the Ki-67 proliferation index. The ROC curves demonstrated that age, rich blood supply to the tumor, and erosion of the dorsum sellae bone can predict the occurrence of a high Ki-67 index. Together, the three risk factors provide a stronger ability to predict the Ki-67 index. The nomogram was developed and validated. Conclusion: Age, rich blood supply to the tumor, and erosion of the dorsum sellae bone are influencing factors for predicting the Ki-67 index. Suitable nomogram prediction models were developed and validated, and there is potential for personalized treatment for PA patients.

## 1. Introduction

Tumors of adenohypophyseal origin are the second most common intracranial tumors in adults with a prevalence rate of 1 in 1000. Most adenohypophyseal tumors are benign and classified as “pituitary adenomas” (PAs) [1]. Although most PAs grow slowly and can be cured by surgery or medications, approximately 50% of PAs are invasive, and some tumors exhibit “aggressive behavior” with rapid growth, recurrence, and resistance to standard treatments [2]. The Ki-67 index is a proliferation indicator of PAs. According to the 2004 World Health Organization (WHO) classification of PAs, a Ki-67 index ≥ 3% indicates invasive growth. Moreover, the WHO 2017 classification of PAs recommends that all patients with PAs must be evaluated for the Ki-67 index, especially when the Ki-67 index is ≥3%, indicating tumor aggression [3]. Refractory PAs are defined as aggressive invasive PAs, one of which is characterized by a high Ki-67 index and requires gross total resection by a neurosurgeon [4]. Gerges et al. reported that a Ki-67 index ≥ 3% may be a useful indicator for close follow up or to justify early prophylactic radiotherapy [5]. Sadeghipour et al. have revealed that a high Ki-67 index is more suitable for predicting invasive PAs [6]. Šteňo et al. have suggested that residual PAs with a Ki-67 index > 2.2% required shorter intervals of follow up with magnetic resonance imaging (MRI) and/or early assistant treatment [7]. Therefore, appropriate prediction of the Ki-67 index may influence the surgical strategy and postoperative follow-up plan for patients with PAs. Brunetti et al. have reported that machine learning analysis of preoperative T2 MRI texture-derived parameters can effectively predict the Ki-67 proliferation index category of pituitary macroadenomas [8]. Bacci et al. demonstrated that the quantitative measurement of the apparent diffusion coefficient value in MRIs could be used to predict the Ki-67 index of PAs [9]. However, the implementation of these prediction methods requires specific software and platforms, and practical clinical applications are difficult to implement. Furthermore, these predictions of Ki-67 index levels are preoperatively speculative, and there are few predictors of Ki-67 index levels based on intraoperative factors. During surgery, the surgeon’s surgical strategy is often adjusted based on intraoperative factors (e.g., tumor texture and blood supply). Therefore, it is necessary to develop a simplified and practical method for predicting the Ki-67 index. Herein, we endeavored to explore and evaluate pre- and intra-operative predictors of the Ki-67 index in patients with PAs.

## 2. Materials and Methods

### 2.1. Patient Population

One hundred and seventy-eight patients who underwent PA resection in the Department of Neurosurgery, The First Affiliated Hospital of USTC, from January 2017 to July 2021 were retrospectively analyzed. The inclusion criteria were as follows: (1) patients undergoing PA resection under microscope or endoscope via transsphenoidal approach; (2) with histopathology-confirmed PAs; (3) with histopathological examination results including the Ki-67 index and levels of pituitary-specific transcription factor, prolactin (PRL), adrenocorticotropic hormone (ACTH), growth hormone (GH), thyroid-stimulating hormone (TSH), follicle-stimulating hormone (FSH), and luteinizing hormone (LH); (4) preoperative pituitary MRI and brain computed tomography (CT) examination (axial + sagittal + coronal) were performed at our hospital. The exclusion criteria were as follows: (1) patients without histopathological examination; (2) with variables that were incompletely collected; (3) preoperative pituitary MRI obtained from outside the hospital. Since this study was retrospective, written informed consent was not required. The research proposal was approved by the Ethics Committee of the First Affiliated Hospital of USTC.

### 2.2. Data Collection

The baseline characteristics included age, sex, postoperative immunohistochemical pituitary hormone positive indices (e.g., FSH, PRL, LH, GH, ACTH, and TSH levels), Knosp grade, tumor breaking through the sellar floor, rich blood supply to the tumor, tumor located inside the sella, erosion of the dorsum sellae bone, and pituitary-specific transcription factor. Specifically, the Ki-67 index refers to the percentage of positive immunohistochemical nuclear staining in all tumor cells. In this study, we used manual method to calculate Ki-67 proliferation index. Specifically, the positive hot-spot area was selected for manual counting and its average value was taken. The Knosp grade was scored by two experienced neurosurgeons based on preoperative pituitary MRI findings. Knosp grades 0, 1, and 2 were regarded as low-grade (<3), and grades 3 and 4 were classified as high-grade (≥3). Tumor tissue breaking through the sellar floor bone was confirmed by intraoperative observation and preoperative CT examination. Rich blood supply to the tumor was determined by two experienced neurosurgeons during the operation and was confirmed by postoperative hematoxylin and eosin (HE) staining, which revealed “chicken-wire” vascular structures. The tumor located inside the sella was defined as the upper margin of the tumor below the line across the anterior and posterior clinoid processes. The erosion and destruction of the dorsum sellae bone were defined as the interruption of the continuity of the dorsum sellae bone in the brain CT. Pituitary-specific transcription factor 1, pituitary cell restricted factor, and splicing transcription factor 1 were immunohistochemically positive, and pituitary-specific transcription factor was definitely positive.

### 2.3. Statistical Analyses

The SPSS17.0 software (SPSS Inc., Chicago, IL, USA) was used for data analyses. Categorical variables were analyzed using the chi-square test, and continuous variables were evaluated using the *t*-test or Mann–Whitney U test. Independent risk factors for Ki-67 index were identified, factors with *p* < 0.05 in univariate analysis were subjected to multivariate analysis, and a predictive receiver operating characteristic curve (ROC) model was drawn. The area under the curve (AUC) was calculated to assess the predictive power of the model, which was classified into three grades: excellent (AUC > 0.8), moderate (AUC 0.7–0.8), and low (AUC 0.6–0.7). Data are expressed as odds ratios (ORs) with 95% confidence intervals (CIs). Statistical significance was set at *p* < 0.05. Multivariate analysis of variance was used to analyze the relevant risk factors, and a regression model was established and converted into a nomogram.

## 3. Results

### 3.1. Patients

**Figure 1:**
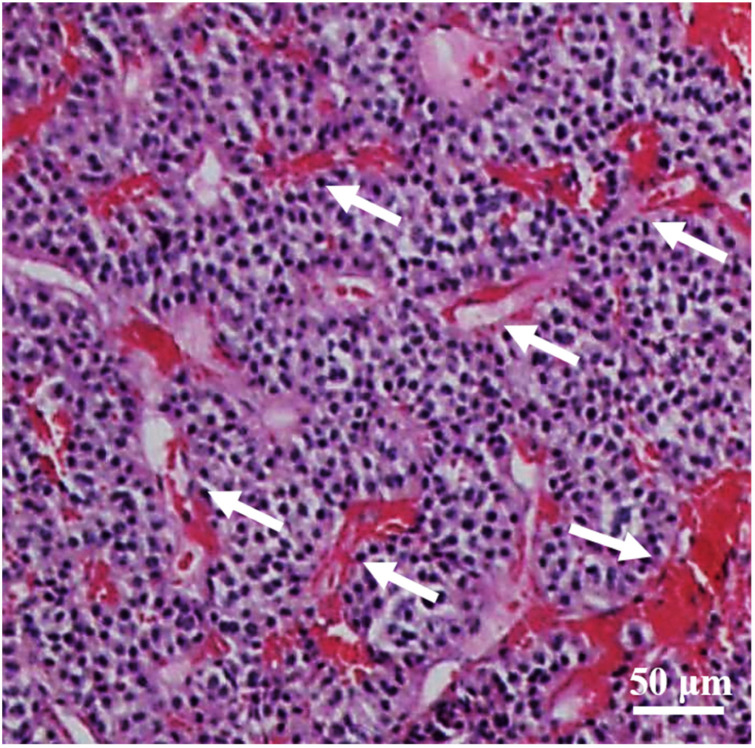
HE staining of a PA, revealing a “chicken-wire” vascular structures (white arrows).

**Figure 2:**
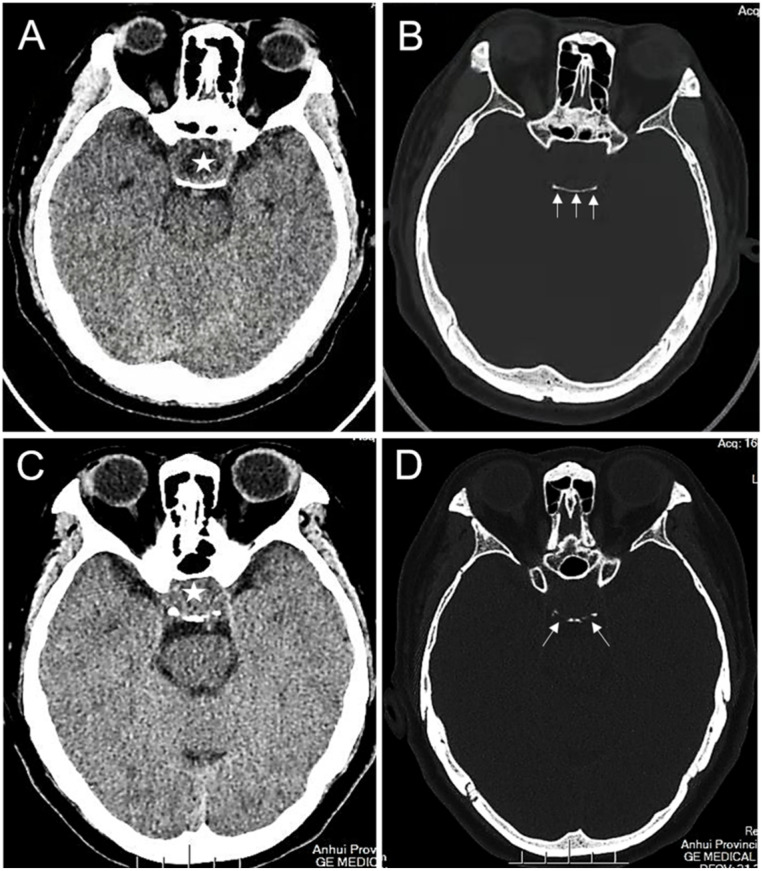
The erosion of the dorsum sellae bone: (**A**,**B**) intrasellar PA (asterisk) with an enlarged sella and an intact dorsum sellae bone (white arrows); (**C**,**D**) Intrasellar PA (asterisk) with an enlarged sella and erosion of the dorsum sellae bone (white arrows).

Altogether, 178 eligible patients with PAs were included in this study and were divided into the Ki-67 < 3% cohort (n = 72) and the Ki-67 ≥ 3% cohort (n = 106). The specific characteristics of the two cohorts are presented in Table 1. Among them, 86 (48.31%) patients were men, and 92 (51.69%) were women, Overall, the mean age of all the patients was 53.08 ± 12.22 years. Of the 178 patients, 44 (24.72%) patients had a Knosp grade ≥ 3, and the tumor broke through the sellar floor in 20 (11.24%) cases. Overall, 111 (62.36%) tumors presented a rich blood supply (Figure 1), and 112 (62.92%) cases indicated erosion of the dorsum sellae bone (Figure 2).

### 3.2. Prediction of Risk Factors Associated with KI-67 Index

The univariate analysis of clinical factors indicated that a Ki-67 index ≥ 3% was significantly associated with age (*p* = 0.0004), rich blood supply to the tumor (*p* < 0.0001), and erosion of the dorsum sellae bone (*p* < 0.0001). No significant correlation was observed between a Ki-67 index ≥ 3% and sex (*p* = 0.499); FSH (*p* = 0.056), LH (*p* = 0.853), PRL (*p* = 0.345), GH (*p* = 0.716), TSH (*p* = 0.343), and ACTH (*p* = 0.516); Knosp grade (*p* = 0.670); tumor breaking through the sellar floor (*p* = 0.489); positive transcription factor (*p* = 0.383). The mean age of Ki-67 index ≥ 3% was 50.46 ± 12.99 years, which was significantly lower than that of the patients with a Ki-67 index < 3% (56.94 ± 9.80) years. Of the 106 PAs with a Ki-67 index ≥ 3%, 84 (79.25%) had a rich blood supply, while only 27 (37.50%) of 72 PAs with a Ki-67 index < 3% had a rich blood supply. Of the patients with a Ki-67 index ≥ 3%, 77.36% (82/106) had erosion of the dorsum sellae bone, which was significantly higher than the 41.67% (30/72) of Ki-67 proliferation index < 3%. In addition, the multivariate regression analysis demonstrated that the rich blood supply to the tumor (OR = 0.124; 95% CI: 0.044–0.355; *p* < 0.001) was an independent risk factor for the Ki-67 index, and the erosion of the dorsum sellae bone (OR = 0.162; 95% CI: 0.057–0.469; *p* = 0.001) was independently related to the Ki-67 index (Table 2). These findings indicate that rich blood supply to the tumor and erosion of the dorsum sellae bone are significant predictors of Ki-67 index ≥ 3%.

**Figure 3:**
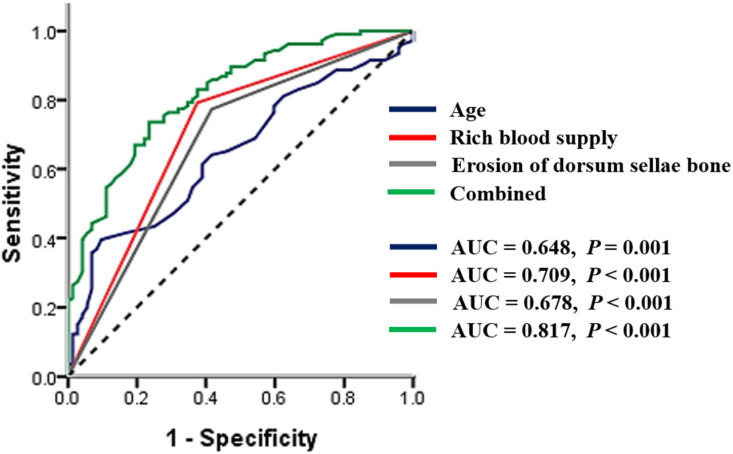
The ROC curve model of three risk factors was obtained by univariate analysis. AUC, area under the curve.

In order to further analyze the predictive value of relevant risk factors on Ki-67 index, ROC curve models were constructed and analyzed (Figure 3). The AUC value of age was 0.648 (95% CI: 0.568–0.728), the rich blood supply was 0.709 (95% CI: 0.629–0.789), and erosion of the dorsum sellae bone was 0.678 (95% CI: 0.596–0.761) (Table 3). The combined AUC value was 0.817 (95% CI: 0.756–0.879), the sensitivity was 73.60%, and the specificity was 76.40%. These results indicated that the combined application of the three factors had a good ability to predict Ki-67 index.

### 3.3. Development and Validation of the Nomogram

**Figure 4:**
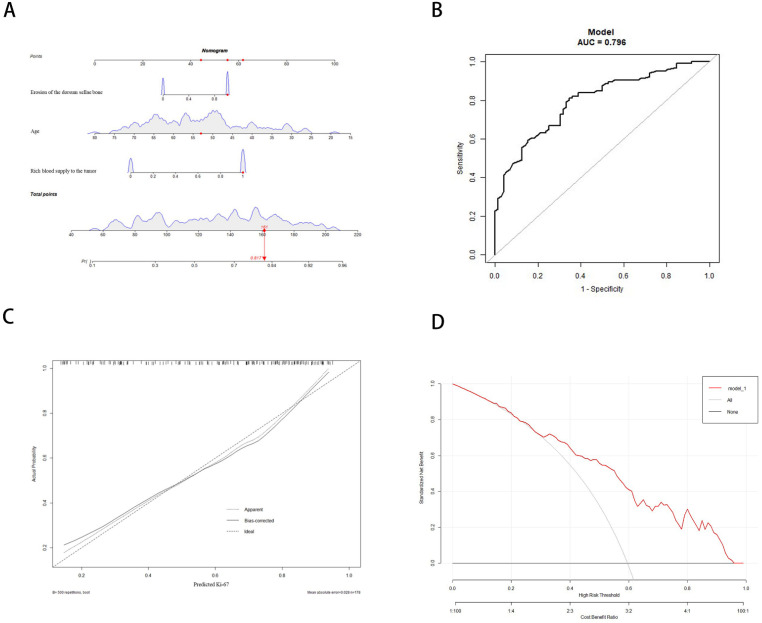
Use of a nomogram to predict the state of Ki-67: (**A**) Clinical and radiological results correspond to a particular point by drawing a straight line up to the point axis. The summary point represents a high (≥3%) probability of Ki-67 when the summary point is located on the total point axis. (**B**) The calibration curve of the model was consistent with the predicted and observed results. (**C**) The ROC curve was constructed to evaluate the nomogram identification ability. (**D**) The ordinate is represented by the net benefit rate, and the abscissa is represented by the high-risk threshold to plot DCA.

Based on the results of multivariate regression analysis, a nomogram model was developed to predict the state of Ki-67 (Figure 4A). A corresponding score was set for each clinical characteristic, and the total score was obtained by plotting a linear point axis, which resulted in a higher Ki-67 probability. Figure 4B indicated that the prediction model had good discriminant power, with an area under the ROC curve of 0.796 (95% CI: 0.731, 0.860). In addition, a bootstrap verification method was used for internal verification of the generated model, and the C index was 0.786. At the same time, the calibration curve was established. Good agreement between predictions and observations was demonstrated in Figure 4C. Taking the net benefit rate as the ordinate and the high risk threshold as the abscissa, the high risk threshold was set at (0.3, 0.9), and the decision curve analysis (DCA) was performed (Figure 4D).

## 4. Discussion

PAs are benign neoplasms originating from anterior pituitary cells, accounting for 10–15% of primary tumors of the central nervous system [10]. In a previous study, although the PAs were benign, some neoplasms still demonstrated invasive or aggressive growth, and tumor recurrence was observed during the postoperative follow up [11]. The Ki-67 index is considered a biomarker of aggressive tumor behavior, which is related to the invasiveness, aggressiveness, and recurrence of PAs [12]. Series studies have shown that a higher Ki-67 index is associated with a higher cell proliferation rate and more aggressive behavior in PAs [13]. Therefore, appropriate prediction of the Ki-67 index can help physicians make better and more personalized treatment strategies for patients with PAs. Here, a Ki-67 index ≥ 3% was considered high, and a Ki-67 index < 3% was classified as low [14]. Trouillas et al. classified pituitary endocrine tumors into five grades according to their invasiveness and proliferative capability, and ki-67 ≥ 3% was an important basis for evaluating tumor proliferative ability. The prognostic value of this grading method for pituitary endocrine tumors has been confirmed [15].

Trott et al. reported that the Ki-67 index was significantly correlated with age in patients with nonfunctional PAs but not with invasiveness [16]. Tanaka et al. reported that the Ki-67 index decreased with age in patients with nonfunctional PAs [17]. Tang et al. established a nomogram model to predict risk factors for postoperative recurrence in patients with nonfunctional PAs and identified advanced age as a significant inhibitor of tumor recurrence [18]. Except for nonfunctional PA, the results of Mohseni et al. revealed that age was negatively correlated with the Ki-67 index level in patients with growth hormone-secreting PAs [19]. Ma et al. have reported that the mean age of the low Ki-67 index group was significantly higher than that of the high Ki-67 index group [20]. Here, the mean age of the patients with a Ki-67 index < 3% was 56.94 ± 9.80 years, which was significantly higher than 50.46 ± 12.99 years of the patients with a Ki-67 index ≥ 3%. Thus, the Ki-67 index level decreased with age in patients with PAs. Notably, statistical analysis demonstrated that age was a risk factor for Ki-67 proliferation index, but not an independent risk factor.

Angiogenesis plays a crucial role in tumor proliferation, and a rich blood supply is a biological characteristic of invasive tumors [21]. Li et al. reported that an increased Ki-67 proliferation index might be a predictor of invasive PAs [22]. Sonoda et al. retrospectively studied the outcomes of 37 cases of residual tumors after surgery for nonfunctional PAs and reported that none of the residual tumors with rich blood supply had spontaneous regression [23]. Morita et al. proposed that tumor angiogenesis was closely related to the invasiveness of PAs, and its marker could predict tumor invasion into the adjacent cavernous sinus [24]. However, studies on the relationship between Ki-67 index and the blood supply to a PA are few. Here, 79.25% of Pas with a Ki-67 index ≥ 3% had a rich blood supply compared to only 37.50% in the Ki-67 index < 3% group. This demonstrated that the existence of a rich blood supply was significantly associated with a high Ki-67 index, which can be used as a potential predictor of the Ki-67 index. Furthermore, the multivariate analysis indicated that a rich blood supply was an independent factor for the Ki-67 index level. These data tell us that if the tumor has an abundant blood supply during surgery, it is likely to indicate that the tumor has a high Ki-67 index., which requires the operator to adjust the surgical strategy to achieve total tumor resection as much as possible, even if there is a significant intraoperative cerebrospinal fluid leak occurring.

The pituitary fossa accommodates the pituitary gland, bounded anteriorly by the tuberculum sellae and posteriorly by the dorsum sellae. Lesions in the pituitary fossa often leads to erosion and thinning of the dorsum sellae bone [25]. Interestingly, PAs in the sellar region can cause destruction of the dorsum sellae bone [26]. In our study, 77.36% of PAs with a Ki-67 index ≥ 3% had erosion of the dorsum sellae bone, which was significantly higher than that of the Ki-67 index < 3% group (41.67%). We hypothesized that this was due to the absorption and destruction of the dorsum sellae bone by tumor cells with a high Ki-67 index during rapid proliferation. This also suggested that the occurrence of dorsum sellae bone erosion was significantly correlated with a high Ki-67 index, and further analysis revealed that erosion of the dorsum sellae bone was an independent factor of the Ki-67 index. Here, the Knosp grade were not correlated with the Ki-67 index, which is consistent with previous studies [12,22].

Finally, an ROC model was plotted to further analyze the reliability of these factors in predicting the Ki-67 index. According to the AUC values, age, and dorsum sellae bone erosion have low predictive accuracy for the Ki-67 index, while the predictive value of rich blood supply is moderate. Significantly, the combination of the three risk factors indicated superior accuracy in predicting a high Ki-67 index. In general, the Ki-67 index is associated with the proliferation, invasion, and aggressiveness of tumor cells. Identifying an appropriate predictor for high Ki-67 index is helpful for devising the surgical plan of patients with PAs and guiding personalized postoperative follow up.

This study had several limitations. First, this study was retrospective and may have been biased in terms of patient selection and data collection. For example, different surgeons may provide different evaluation criteria for observation targets, which may reduce the reliability of the results. Second, our data were from a single center, the sample size of the study was limited, and some data may be lost during patient follow up, which limits the ability to identify potential risk factors for the Ki-67 index. Finally, preoperative treatment with growth inhibitor analogs or dopamine agonists in a small number of patients can affect PA cell growth as well as Ki-67 expression and tumor tissue characteristics. Therefore, further prospective, large-sample, multicenter studies are needed to validate our results.

## 5. Conclusions

In conclusion, we found that age, erosion of the dorsum sellae bone, and rich blood supply of PA may play a certain role in predicting higher Ki-67 index. In addition, we developed and validated a suitable nomogram prediction model that has the potential value of further assisting surgeons in developing better and more personalized treatment strategies for patients with PA.

## Figures and Tables

**Table 1:** Clinical characteristics of patients with PAs in the Ki-67 index < 3% and Ki-67 index ≥ 3% cohorts.

Variables	Ki-67 < 3% (n = 72)	Ki-67 ≥ 3% (n = 106)	t/χ^2^	*p*-Value
Age (year)	56.94 ± 9.80	50.46 ± 12.99	3.594	0.0004
Sex				
Male	37 (51.39%)	49 (46.23%)	0.458	0.499
Female	35 (48.61%)	57 (53.77%)		
FSH				
Yes	34 (47.22%)	35 (33.02%)	3.644	0.056
No	38 (52.78%)	71 (66.98%)		
LH				
Yes	13 (18.06%)	35 (33.02%)	0.034	0.853
No	59 (81.94%)	88 (83.02%)		
PRL				
Yes	19 (26.39%)	35 (33.02%)	0.892	0.345
No	53 (73.61%)	71 (66.98%)		
GH				
Yes	14 (19.44%)	23 (21.70%)	0.132	0.716
No	58 (80.56%)	83 (78.30%)		
TSH				
Yes	5 (6.94%)	4 (3.77%)	0.898	0.343
No	67 (93.06%)	102 (96.23%)		
ATCH				
Yes	6 (8.33%)	12 (11.32%)	0.421	0.516
No	66 (91.67%)	94 (88.68%)		
Knosp grade				
<3	53 (73.61%)	81 (76.42%)	0.181	0.670
≥3	19 (26.39%)	25 (23.58%)		
Tumor breaking through sellar floor				
Yes	6 (8.33%)	14 (13.21%)	1.021	0.312
No	66 (91.67%)	92 (86.79%)		
Rich blood supply to the tumor				
Yes	27 (37.50%)	84 (79.25%)	31.830	<0.0001
No	45 (62.50%)	22 (20.75%)		
Tumor located inside the sella				
Yes	26 (36.11%)	33 (31.13%)	0.480	0.489
No	46 (63.89%)	73 (68.87%)		
Erosion of the dorsum sellae bone				
Yes	30 (41.67%)	82 (77.36%)	23.41	<0.0001
No	42 (58.33%)	24 (22.64%)		
Positive of transcription factor				
Yes	56 (77.78%)	88 (83.02%)	0.762	0.383
No	16 (22.22%)	18 (16.98%)		

PAs, pituitary adenomas; FSH, follicle-stimulating hormone; LH, luteinizing hormone; PRL, prolactin; GH, growth hormone; TSH, thyroid-stimulating hormone; ACTH, adrenocorticotropic hormone.

**Table 2:** Multivariate regression analysis of factors related to Ki-67 index.

Factors	OR	95% CI	*p*-Value
Age	0.294	0.078–1.612	0.228
Rich blood supply to the tumor	0.124	0.044–0.355	0.000
Erosion of the dorsum sellae bone	0.162	0.057–0.469	0.001

OR, odds ratio; CI, confidence interval.

**Table 3:** The AUC values in ROC curves.

Factors	AUC	SE	95% CI
Age	0.648	0.041	0.568–0.728
Rich blood supply to the tumor	0.709	0.041	0.629–0.789
Erosion of the dorsum sellae bone	0.678	0.042	0.596–0.761
Combined	0.817	0.031	0.756–0.879

AUC, area under the curve; CI, confidence interval; SE, standard error; ROC, operating characteristic curve.

## Data Availability

The original contributions presented in the study are included in the article, further inquiries can be directed to the corresponding author.
